# Developmental exposure window influences silver toxicity but does not affect the susceptibility to subsequent exposures in zebrafish embryos

**DOI:** 10.1007/s00418-020-01933-2

**Published:** 2020-10-21

**Authors:** Paige C. Robinson, Hannah R. Littler, Anke Lange, Eduarda M. Santos

**Affiliations:** 1 Biosciences, College of Life and Environmental Sciences, Geoffrey Pope Building, University of Exeter, Exeter, EX4 4QD UK; 2 Centre for Environment, Fisheries and Aquaculture Science, Barrack Road, The Nothe, Weymouth, DT4 8UB Dorset UK; 3 Sustainable Aquaculture Futures, University of Exeter, Exeter, EX4 4QD UK

**Keywords:** Pulsed exposure, Repeated exposure, *Danio rerio*, Teleost, Sensitivity

## Abstract

Silver is a non-essential, toxic metal widespread in freshwaters and capable of causing adverse effects to wildlife. Its toxic effects have been studied in detail but less is known about how sensitivity varies during development and whether pre-exposures affect tolerance upon re-exposure. We address these knowledge gaps using the zebrafish embryo (*Danio rerio*) model to investigate whether exposures encompassing stages of development prior to mid-blastula transition, when chorion hardening and epigenetic reprogramming occur, result in greater toxicity compared to those initiated after this period. We conducted exposures to silver initiated at 0.5 h post fertilisation (hpf) and 4 hpf to determine if toxicity differed. In parallel, we exposed embryos to the methylation inhibitor 5-azacytidine as a positive control. Toxicity increased when exposures started from 0.5 hpf compared to 4 hpf and LC50 were significantly lower by 1.2 and 7.6 times for silver and 5-azacyitidine, respectively. We then investigated whether pre-exposure to silver during early development (from 0.5 or 4 hpf) affected the outcome of subsequent exposures during the larvae stage, and found no alterations in toxicity compared to naïve larvae. Together, these data demonstrate that during early development zebrafish embryos are more sensitive to silver when experiments are initiated at the one-cell stage, but that pre-exposures do not influence the outcome of subsequent exposures, suggesting that no long-lasting memory capable of influencing future susceptibility was maintained under our experimental conditions. The finding that toxicity is greater for exposures initiated at the one-cell stage has implications for designing testing systems to assess chemical toxicity.

**Electronic supplementary material:**

The online version of this article (10.1007/s00418-020-01933-2) contains supplementary material, which is available to authorized users.

## Introduction

Silver is a non-essential, toxic metal capable of causing damage to biodiversity in aquatic systems worldwide. Growing applications of silver nanoparticles in industrial processes (Thamilselvi and Radha 2017) and the resulting increase in contamination of the environment have made silver an increasingly important pollutant to study. The bioavailability and toxicity of silver vary greatly depending on environmental conditions (Paquin and Di Toro 2008), life stage (Groh et al. 2015), and mode of uptake (Hogstrand and Wood 1998). However, the precise mechanisms underpinning variation in toxicity remain understudied. Understanding the determinants of susceptibility and the impact that multiple exposures have on individuals and populations is important for the protection of ecosystems.

The predominant sources of silver pollution have varied over the last few decades. Initial concerns related to the discharge of silver by the photographic industry in the twentieth century, with concentrations reaching up to 1 g/kg in soils and 38 μg/L in aquatic environments near mining sites (Purcell and Peters 1998). Legislation to limit pollution in Europe, Australia and New Zealand defined a safe limit for ionic silver as 0.05 µg/L, while limits are 0.1 µg/L and 3.2 µg/L for Canada and the USA, respectively (Kwak et al. 2016). These regulations led to concentrations being substantially reduced in the environment (Juncos et al. 2017). Recently, the broad utility of silver nanoparticles as a catalytic agent in green synthesis (Razack et al. 2016) and as an antibacterial agent in the textile industry (Dubas et al. 2006), wound healing (Kumar et al. 2018) and water treatment (Chen et al. 2017; Ngoc Dung et al. 2019) have led to an increase in the concentrations of silver in aquatic systems. As the most commercialised nanomaterial worldwide (Valerio-García et al. 2017), around 500 tonnes of silver nanoparticles are produced each year (Kwak et al. 2016), and once they enter aquatic environments, silver ions on the nanoparticle surface can dissolve over time, increasing the toxic effect on organisms (Garcia-Reyero et al. 2015). Recorded silver ion levels recently ranged from 40 to 320 ng/L in the river Rhine, Germany (McGillicuddy et al. 2017), and concentrations are expected to continue to increase. Once silver enters the environment, it can occur in a variety of molecular species depending on other environmental variables, including pH, salinity, and presence of organic matter in the water, resulting in varying toxicity. Biotic Ligand Models (BLMs) incorporate this information and are used to estimate toxicity in a given environment (Paquin and Di Toro 2008).

Freshwater fish are particularly vulnerable to silver as it is transported into gill ionocytes via sodium ion channels. Once inside the cell, it binds at Mg^2+^ binding sites on the cytoplasmic side of the basolateral Na^+^–K^+^ ATPase pump, preventing ATP hydrolysis and inhibiting sodium and chloride transport between the gill ionocyte and the blood plasma (Bury et al. 1999). As gills are the main site of osmoregulation, the loss of ions from blood plasma can cause circulatory collapse of fluid volume regulation due to increased blood viscosity and increased pressure on the cardiovascular system. Ion transport interruption can also cause an increase in intracellular acidity which can initiate a branchial inflammatory response, increasing mucus production and interrupting gas transfer. This respiratory inhibition is less severe than the osmoregulatory effect, but can be lethal if silver concentration causes damage to the gill epithelium and blood oxygen tension drops (Hogstrand and Wood 1998). Once silver crosses the gill epithelium and enters the blood stream, it is transported by macroglobulins, such as ceruloplasmin, hijacking the transport mechanism for essential metals (Hanson et al. 2001). Silver is then deposited in hepatic and renal cells among others, where metallothionein and other metal chaperones bind to inactivate and transport silver for excretion (Lansdown 2006). Hepatic excretion through exocytosis into the bile is the main pathway for silver removal with renal excretion through blood filtration also contributing to removal of silver ions from the blood stream (Kleiven et al. 2018).

During embryonic development in fish, silver exposure and uptake differ from that observed in adults due to the absence of functional gills and to chorion permeability changes during the first few hours post fertilisation (hpf). Silver has been observed throughout embryo tissues when concentrations exceed 30 µg/L (Böhme et al. 2017). Before the development of functioning gills, ionocytes across the epithelial surface are the main site of ion regulation (Fu et al., 2010) and gas exchange (Wells and Pinder 1996). Embryos are likely to be more sensitive than adult fish to silver exposure due to their incomplete development and suboptimal protective mechanisms (Groh et al. 2015). For exposures initiated during the first two hours of zebrafish development, silver concentrations within embryo tissues were observed to be up to 20 times higher compared to exposures later in development (Böhme et al. 2015). This has been linked to the permeability of the chorion during this early developmental stage as it swells adapting to the change in osmotic pressure post fertilisation (Peterson and Martin-Robichaud 1982). Once the chorion has hardened by 4 hpf, it has been shown to act as a protective barrier against silver uptake in zebrafish embryos (Guadagnolo et al. 2001). Silver ions have also been shown to accumulate within oocytes in exposed adult females, further increasing the exposure concentration, and thus sensitivity during early life stages, although most of the recorded silver accumulation was located in chorion structures (Bӧhme et al. 2017).

Similarly to other toxic metals, silver can cause oxidative damage to DNA, lipids and proteins due to acting as a catalyst to increase the production of reactive oxygen species (Vandegehuchte and Janssen 2011). Silver also alters the functioning of multiple molecular pathways including oxidative phosphorylation (van Aerle et al. 2013), immune response (Garcia-Reyero et al. 2015), cell cycling (Kang et al. 2016), and calcium signalling (Xu et al. 2018). Metallothionein upregulation was observed in 72 and 96 hpf zebrafish embryos after 24 h of silver exposure prior to sampling (Boyle and Goss 2018), and genes related to olfactory bulbs and lateral line neuromasts, as well as skin ionocytes, were also affected when exposures were initiated at 1 hpf (Osborne et al. 2016). Silver has also been shown to cause epigenetic modifications in fish, which can alter gene transcription long after the exposure has ended (Xu et al. 2018).

In freshwater ecosystems, pollutant concentrations often fluctuate rapidly over time, resulting in intermittent exposures (Handy 1994), which can alter organism response due to toxicodynamic recovery (Ashauer et al. 2017). Despite this, the impacts of variable exposure conditions on individuals and populations have rarely been studied, and this is a priority for study, especially for exposures occurring at vulnerable life stages (Plautz and Salice 2013). Experiments conducting repeated exposures for various chemicals have resulted in either increased or decreased sensitivity when organisms were re-exposed compared to the naïve response. When exposed to 17α-ethinyloestradiol during the first 48 h of development, zebrafish show increased sensitivity to subsequent oestrogen exposures later in development (Green et al. 2018), and similar results were observed following intermittent exposures to 17α-ethinyloestradiol in roach (Lange et al. 2009). Higher concentrations of fenvalerate were recorded in intermittently exposed trout as opposed to those continuously exposed (Curtis et al. 1985), and pulsed exposures to copper during early development were also shown to be more toxic than a continuous exposure at the same concentration (Boyle et al. 2020). However, if adult fish were given time to acclimate to near lethal copper concentrations, an increase in tolerance was observed (Anadu et al. 1989), usually attributed to increased metallothionein expression (Bradley et al. 1985). Genetic variation in populations may partially contribute towards observed tolerance to metal exposure as observed in earthworms (Kille et al. 2013); however, epigenetic DNA methylation alterations in response to altered conditions have also been observed to increase tolerance in stickleback after marine to freshwater movement (Artemov et al. 2017), and human lympoblast cells after acclimation to cadmium (Ye et al. 2014). More work is needed to understand the impact of repeated exposures on organisms at the physiological and molecular level.

The timing of initial exposure during embryo development is also important to consider as it has been shown to be crucial in the toxicity of various chemicals, including silver, due to the dynamic nature of the processes occurring during early developmental stages (Böhme et al. 2015; Groh et al. 2015). During the first four hours of zebrafish development, rapid cell proliferation and epigenetic reprogramming occur to allow for cell specification, uniform chromatin architecture and the removal of deleterious markers (Jiang et al. 2013; Potok et al. 2013; Skvortsova et al. 2018). At this time, it is hypothesised that the epigenome is more susceptible to alterations due to stressor exposure, potentially increasing the toxicity of stressors capable of disrupting it (Gellert and Heinrichsdorff 2001). This is supported by data for the methylation inhibitor 5-azacytidine which has been shown to alter zebrafish methylation and cause developmental abnormalities when exposures occur during the reprogramming period at 2–3 hpf; however, toxicity is significantly reduced when exposures occur after reprogramming is complete (> 6 hpf; Martin et al. 1999; Kamstra et al. 2017). Zygotic genome activation (ZGA) also occurs during early development at the mid-blastula transition (Pálfy et al. 2017), after which embryos are able to more readily modify transcript abundance and protein synthesis in response to chemical exposure, providing another hypothesis to explain potential changes in sensitivity to chemical exposure during early development (reviewed in Schulz and Harrison 2019). The impact of exposures during these potential periods of sensitivity, and in particular, the effects of these exposures on the susceptibility of embryos to subsequent exposures remains poorly understood.

Here, we hypothesise that the developmental period encompassing epigenetic reprogramming will be particularly sensitive to silver exposures and most likely lead to long-lasting effects and changes in susceptibility upon re-exposure due to alterations in the epigenome. To examine these hypotheses, we conducted exposures to silver (Ag^+^), using the zebrafish embryo model, initiated at two periods during early development (prior to, and after, epigenetic reprogramming, chorion hardening and ZGA) to determine if the timing of initiation of exposure caused measurable changes in silver toxicity. The methylation inhibitor 5-azacytidine was used as a positive control in parallel due to its known methylation inhibitor effects during zebrafish development (Christman 2002). Further, we conducted exposures later in development with naïve and pre-exposed embryos to determine if pre-exposures during the critical windows of sensitivity defined above influenced susceptibility to future exposures.

## Materials and methods

### Chemicals

All chemicals were purchased from Sigma-Aldrich, Gillingham UK, unless otherwise stated, and working stock solutions were made in ultrapure water and used within two weeks.

### Zebrafish husbandry and embryo collection

Adult WIK zebrafish stock populations used for embryo collection originated from a breeding population maintained at the University of Exeter and were kept under the conditions described in Paull et al. (2008). Adults were kept in 8 L tanks supplied with flow through standardised synthetic freshwater (according to OECD guidelines; ISO-7346/3 guideline), aerated and heated to 28 °C. Fish were kept in a 12:12 light:dark cycle with gradual dawn and dusk transitions of 30 min, and fed live *Artemia* once daily and GEMMA Micro pellet food, Skretting USA, twice daily.

For embryo collection, adults were randomly allocated to 20 2:1 female:male spawning chambers (3 fish per chamber) overnight with a divider separating males and females. At dawn, the divider was removed and fish were allowed to spawn for 10 min before embryo collection and, because of welfare considerations, fish were kept in spawning chambers for a minimum period of time. Embryos were pooled to avoid any potential bias and 50 embryos were then randomly allocated into each exposure dish containing 50 ml of water at the appropriate exposure conditions prior to 30 min post fertilisation (0.5 hpf). For exposures starting at 4 hpf, embryos were allocated to 50 ml of control water, with chemicals added at 4 hpf.

### Impact of timing of initial exposure to silver or 5-azacytidine on toxicity for zebrafish embryos

To test whether the sensitivity of embryos to silver exposure differed with the developmental stage at which exposures were initiated, we performed 48 h dose–response curves initiated at 0.5 hpf or 4 hpf (including or excluding the period of epigenetic reprogramming and the period of chorion hardening). All chemical exposures for both dose-response curves were performed in triplicate with appropriate concentrations of each chemical added in 50 ml aerated ISO water (according to the ISO-7346/3 guideline, ISO water diluted 1:5) with the temperature maintained at 28 °C. Data were collected from three independent experiments, with each experiment using a single pool of embryos to generate full dose-response curves initiated at 0.5 and 5 hpf (ensuring reduced technical variability between individual dishes within the same experiment and that the three experiments were independent biological replicates). The range of concentrations tested was chosen to cover a full mortality curve, based on preliminary experiments (data not shown). For 0.5–48.5 hpf dose-response curves, embryos were exposed to nine concentrations of silver, added in the form of silver nitrate, ranging from 0 to 60 µg/L Ag^+^, or 5-azacytidine as a positive control, ranging from 0 to 35 mg/L. Unfertilised embryos were identified by observation under a light microscope (Nikon, Japan) and removed at 3 hpf, and the number of embryos was adjusted to 20 per dish. At this time point, the stage of development was confirmed to be consistent for all embryos.

**Fig. 1:**
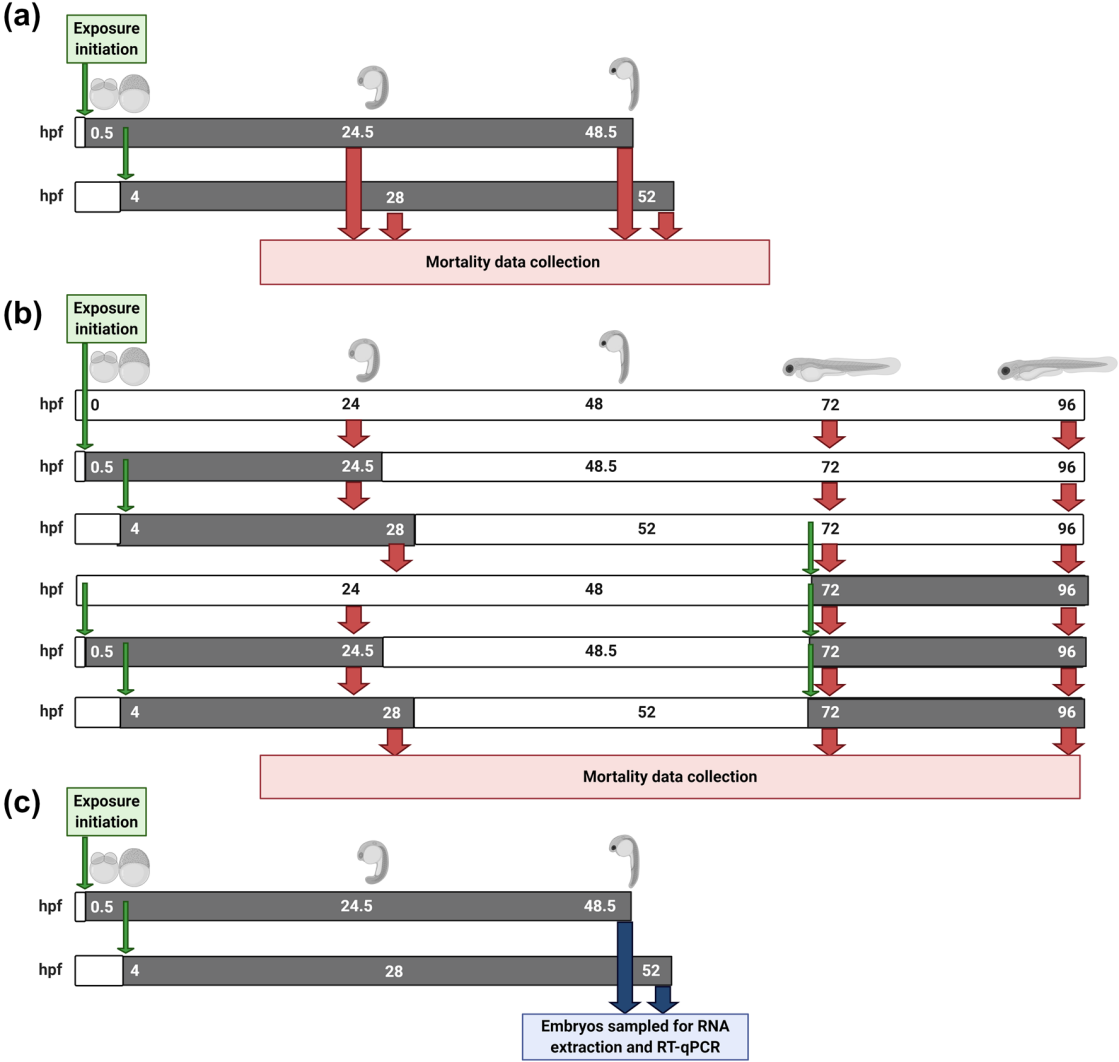
Schematic of the experimental design for the experiments included in this study. **a** Dose-response curves for silver and 5-azacytidine when exposures were initiated at 0.5 or 4 hpf. **b** Repeated exposure experiments for silver to investigate if pre-exposures initiated at 0.5 and 4 hpf influence the susceptibility of zebrafish embryos upon re-exposure. **c** Embryo exposure experiments to investigate the effects of exposure to silver and 5-azacytidine on transcription of target genes. Exposure periods are shown in grey bars, with clean water periods shown in white bars. Exposure medium was replaced every 24 h for every embryo pool, with the first replacement delayed until 24 h after exposure initiation. Green arrows represent the points of initiation of exposures, red arrows represent the points at which mortality was assessed and blue arrows represent the points where embryo samples were collected for RNA extraction. Schematics were created with BioRender.com

For 4–52 hpf dose–response curves, pools of 20 fertilised embryos were exposed from 4 hpf to 9 concentrations of silver added in the form of silver nitrate, ranging from 0 to 80 µg/L Ag^+^, or 5-azacytidine, ranging from 0 to 185 mg/L, for 48 h. Mortality and developmental endpoints were recorded after 24 and 48 h of exposure. The exposure water containing the appropriate concentration of silver or 5-azacytidine was replaced 24 h after exposure initiation and any dead embryos were recorded and removed. Unexposed embryos were maintained and assessed in parallel under the same conditions for each exposure period as a negative control. A schematic description of methods is given in Fig. 1a.

### Influence of pre-exposure to silver during early development on the susceptibility of larvae upon re-exposure

To determine if pre-exposure to silver caused altered tolerance upon re-exposure, initial exposures were performed as described previously for dose-response curves, with a concentration of 30 µg/L Ag^+^ (defined as approximately the LC20 for the most sensitive period of exposure (0.5–48.5 hpf) and sufficiently high to induce adverse effects on exposed embryos). Exposures were initiated at 0.5 or 4 hpf for 24 h, before rinsing embryos and maintaining them in control conditions for a depuration period until 72 hpf. At this point, larvae from control, 0.5–24.5 and 4–28 hpf exposure groups were exposed to 30 µg/L Ag^+^ for 24 h. This concentration was chosen as it caused over 50% mortality in naïve larvae after a 24 h exposure initiated at 72 hpf, so any alteration in tolerance due to a pre-exposure before or after the epigenetic reprogramming period could be determined. Mortality and developmental endpoints were recorded at 48.5 and 52 hpf, and again at 72 and 96 hpf, and dead embryos were removed from dishes upon examination. Embryos from the three initial exposure conditions were maintained in parallel without undergoing a re-exposure as a negative control. For each treatment group, 6 dishes containing 20 embryos were included (*n* = 6 biological replicates per treatment). A schematic description of these methods is given in Fig. 1b.

### Transcript profiling

Real-time quantitative PCR (RT-QPCR) was used to quantify the transcription of target genes in zebrafish embryo pools after a 48 h exposure to silver and to 5-azacytidine. To do this, six replicate embryo pools (*n* = 20) were exposed to LC10 concentrations of silver (added in the form of silver nitrate, 25 µg/L Ag^+^) or 5-azacytidine (7.85 mg/L) for 48 h, initiated at 0.5 or 4 hpf. These concentrations were chosen to allow for investigation of potential mechanisms of action of the chemicals of interest rather than identifying biomarkers of overt toxicity. Embryo pools were then flash-frozen in liquid nitrogen and stored at − 80 °C. A schematic description of these methods is given in Fig. 1c. The following transcripts were selected for analysis: *catalase* (*cat*), a biomarker for oxidative stress (Yeo and Kang 2008); *metallothionein 2* (*mt2*) a biomarker of metal exposure (Kägi and Schäffer 1988); and *DNA (cytosine-5-)-methyltransferase 1* (*dnmt1*) and *DNA (cytosine-5-)-methyltransferase 3bb.2* (*dnmt3bb.2*), which encode for enzymes responsible for cytosine methylation maintenance and de novo methylation (Campos et al. 2012), respectively. Primers for each target gene were designed with Beacon Designer 3.0 software (Premier Biosoft International, Paulo Alto, CA) using zebrafish NCBI Ensembl sequences, and purchased from Eurofins Genomics, Ebersberg Germany (Table 1). Primer specificity throughout the range of detection was confirmed by the observation of single amplification products of the expected size and melt temperature (*T*_m_), and optimised by performing a standard curve for each primer pair as previously described (Laing et al. 2016). Over the detection range, the linear correlation (*R*^2^) between the mean Ct and the logarithm of the cDNA dilution was > 0.86 in each case, and efficiencies were between 97.5 and 120.5. The primer sequences, PCR product sizes, annealing temperatures and PCR efficiencies for each primer pair are shown in Table 1.

**Table 1:** Target genes, Ensembl gene IDs, gene symbols, primer sequences, amplicon product sizes, annealing temperatures (*T*_a_), and PCR efficiencies for the qPCR assays used in this study

Gene name	Ensembl gene ID	Gene symbol	Forward primer (5′-3′)	Reverse primer (5′-3′)	Product size (bp)	*T*_a_ (°C)	PCR efficiency (%)
*ribosomal protein L8*	ENSDARG00000014867	*rpl8*	CCGAGACCAAGAAATCCAGAG	CCAGCAACAACACCAACAAC	91	57.0	103.8
*catalase*	ENSDARG00000104702	*cat*	AGTTCCCTCTGATTCCTGTG	ATGGCGATGTGTGTCTGG	173	61.0	116.3
*metallothionein 2*	ENSDARG00000041623	*mt2*	AATGTGAATCTGTTTGTCTACTCC	GCATCGTTTTCCCTCTTTAGC	164	61.5	97.5
*DNA (cytosine-5)- methyltransferase 1*	ENSDARG00000030756	*dnmt1*	CGCTGTCGTGTTGAGTATGC	TCCCTTGCCCTTTCCTTTCC	180	58.0	120.5
*DNA (cytosine-5)- methyltransferase 3bb.2*	ENSDARG00000057830	*dnmt3bb.2*	TGATGCCGTGAAAGTGAGTC	TTGCCGTGTAGTGATAGTGC	172	58.5	109.5

RNA was extracted from six embryo pools of *n* = 20 from each treatment group using the AllPrep DNA/RNA mini kit (Qiagen, Hilden, Germany), according to the manufacturer’s instructions. RNA concentration and purity were assessed with a NanoDrop ND-1000 Spectrophotometer (NanoDrop Technologies, Wilmington, USA). cDNA was synthesised according to manufacturer's instructions from 1 µg of total RNA treated with RQ1 DNase (Promega, Southampton, UK) using random hexamers (Eurofins Genomics) and M-MLV reverse transcriptase (Promega). cDNA was diluted 1:4 and RT-QPCR was performed in duplicate in an iCycler iQ Real-time Detection System (Bio-Rad Laboratories, Hercules, CA) using SYBR Green chemistry as described previously (Laing et al. 2016). A template-minus negative control was run in duplicate on each plate to verify the absence of DNA contamination. Efficiency-corrected relative expression levels were determined by normalising to a control gene, ribosomal protein l8 (*rpl8*), which was previously shown to have consistent expression in zebrafish embryos, including during metal exposures (van Aerle et al. 2013; Fitzgerald et al. 2016), and was found to be stable across all treatment groups (analysis of variance for cycle treshold (Ct) values showed no differences between groups; *p* = 0.394).

### Data analysis

Mortality curves for silver and 5-azacytidine were generated in R (https://www.r-project.org/) using the drc package to run a dose–response model (drm) (Christian Ritz and Jens C. Streibig, drc R cran). To test significant differences between mortality curve models at 0.5 hpf and 4 hpf for each chemical and whether an interaction occurred, two-way ANOVAs were run in R. The dose–response models were used to calculate the lethal concentrations (LC) responsible for 10, 20, 50 and 90% mortality after 48 h of exposure. These concentrations were used to inform the choice of exposure concentrations for subsequent experiments.

For re-exposure studies, barplots of average percentage mortality within each embryo pool were plotted in R (± SE) using ggplot2, and a Kruskal–Wallis rank-sum test was performed in R, given that the data did not meet the assumptions of normality and equal variance required for parametric tests. When significant differences were identified, pairwise comparisons using Wilcoxon rank-sum test were performed to identify significant differences between treatment groups.

All gene transcription data were first scrutinised using the Chauvenet’s criterion to detect outliers and these biological replicates (a maximum of one per treatment group) were subsequently removed before analysis (Chauvenet 1863). For each gene, data were tested for normality (Shapiro–Wilk test) and heterogeneity (Levene test) before implementing statistics. If data were found not to fit with these assumptions for parametric tests, these were then log-transformed. Transformed data were again tested for normality and equal variance and if at either stage data were normally distributed, an ANOVA and Tukey’s post hoc test were performed to identify all pairwise differences between treatment groups. If even after log-transforming the data, these were not normally distributed, a non-parametric Kruskal–Wallis test was performed on the non-log-transformed data followed by Wilcoxon rank-sum post hoc test to identify differences between exposed embryos and controls. A separate model for each gene was used to test effects on gene transcription as a result of exposure to silver or 5-azacytidine. All data were considered statistically significant when *p* < 0.05.

## Results

### Impact of timing of initial exposure to silver or 5-azacytidine on toxicity for zebrafish embryos

**Fig. 2:**
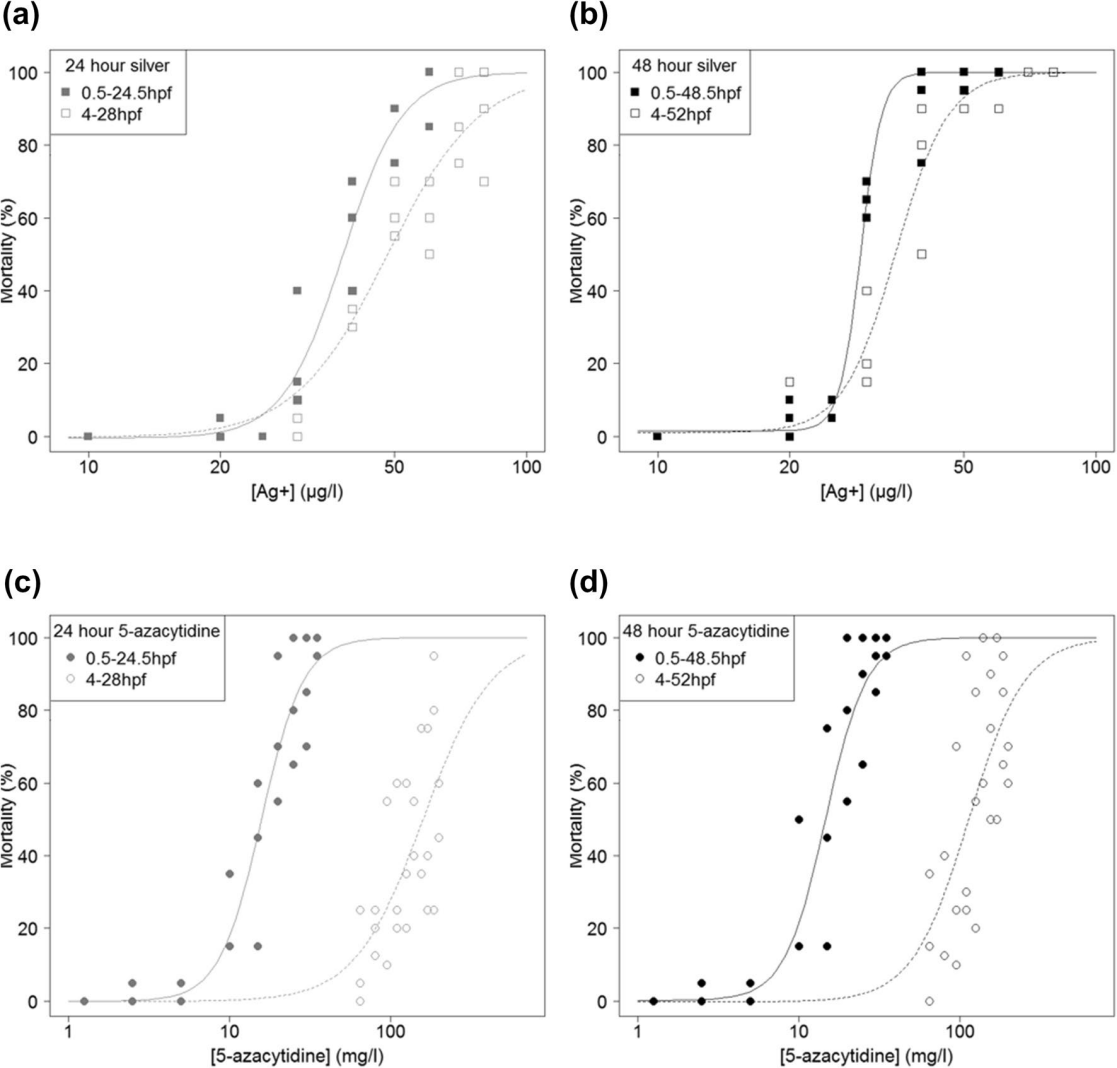
Cumulative embryo mortality curves resulting from exposure to silver or 5-azacytidine. Comparisons between mortality curves for **a** silver exposures initiated at 0.5 and 4 hpf, after 24 h of exposure, **b** silver exposures initiated at 0.5 and 4 hpf, after 48 h of exposure, **c** 5-azcytidine exposures initiated at 0.5 and 4 hpf, after 24 h of exposure and **d** 5-azacytidine exposures initiated at 0.5 and 4 hpf, after 48 h of exposure. Each point represents the percentage of mortality in one replicate dish containing 20 embryos, with three independent replicates per exposure concentration. A dose-response model with four-parameter log-logistic function was fitted to produce each curve in R using the drc package. Statistical analysis of the difference between mortality curves at each exposure period are given in Table 2

The developmental stage at which exposures were initiated caused significant differences in silver toxicity after 24 and 48 h of exposure (Fig. 2a and b; Table 2). Greater toxicity was observed for exposures initiated at 1-cell stage (0.5 hpf, prior to the period of epigenetic reprogramming, chorion hardening and ZGA), compared to when exposures were initiated at sphere stage (4  hpf, after the main period of epigenetic reprogramming, chorion hardening and the initiation of ZGA) when mortality was assessed at 24 h after the initiation of the exposure (Fig. 2a, *p* = 0.0227; Table 2), but the increase in toxicity was small. LC50 concentrations derived from the drc model were 29 µg/L Ag^+^ for the exposure period commencing at 0.5 hpf, and 35 µg/L Ag^+^ for the 4–52 hpf exposure period (nominal concentrations; Table 3) corresponding to an increase in toxicity of 1.2-fold. A significant interaction between exposure period and toxicity was also recorded, where the exposure initiated at 0.5 hpf caused a steeper mortality curve than exposure initiated at 4 hpf (Fig. 2a and b, *p* = 0.0119 and 0.0243 for mortality data collected 24 h and 48 h after the onset of the exposure; Table 2). In addition, we compared the mortality data obtained for 24 h and 48 h to assess whether mortalities increased progressively over time. Mortality continued to increase after 24 h of exposure and was significantly greater at 48 h compared to 24 h both for exposures initiated at 0.5 and 4 hpf (*p* = 0.019 and 0.00021 for exposures initiated at 0.5 and 4 hpf, respectively; Fig. S1a and b; Table S1).

**Table 2:** Analysis of variance models for the relationships between exposure concentrations, time at which exposures were initiated (0.5 or 4 hpf) and the interaction between the two variables

ANOVA						
	Concentration		Exposure initiation (0.5 or 4 hpf)		Interaction	
	*F*	*p*	*F*	*p*	*F*	*p*
(a) Silver						
24 h	198.582	< 2e−16***	5.580	0.0227*	6.884	0.0119*
48 h	211.925	< 2e−16***	2.592	0.1146	5.443	0.0243*
(b) 5-azacytidine						
24 h	14.64	0.000335***	64.14	8.43e−11***	114.31	4.81e−15***
48 h	35.47	1.90e−07***	42.81	2.11e−08***	81.17	2.04e−12***

Zebrafish embryos were exposed to (a) silver or (b) 5-azacytidine and mortality was recorded after 24 and 48 h of exposure. The resulting *F* and *p* values are shown for each model. (Significance codes: ∗ *p* < 0.05, ∗  ∗ *p* < 0.01, ∗  ∗  ∗ *p* < 0.001). Graphical depictions of data shown in Fig. 2

**Table 3:** Comparison of the lethal concentrations (LC) ± standard error of the mean following exposure of zebrafish embryos to silver and 5-azacytidine for 48 h, initiated at 0.5 or 4 hpf (one-cell stage or sphere stage, respectively)

Proportion of mortality after 48 h exposure (%)	Silver LC (µg/L) ± SEM		5-azacytidine LC (mg/L) ± SEM	
	0.5–48.5 hpf	4–52 hpf	0.5–48.5 hpf	4–52 hpf
LC10	25.722 ± 1.273	25.823 ± 1.662	7.857 ± 1.782	46.612 ± 12.401
LC20	26.905 ± 0.950	28.878 ± 1.403	9.922 ± 1.710	64.629 ± 12.832
LC50	29.056 ± 0.419	34.962 ± 1.013	14.787 ± 1.425	112.997 ± 11.994
LC90	32.822 ± 1.135	47.336 ± 2.300	27.828 ± 3.975	273.929 ± 49.337

Lethal concentrations were determined from the dose-response models with four-parameter log-logistic function ran in R using package drc, and based on nominal chemical concentrations

The developmental stage at which exposures were initiated caused pronounced significant differences in toxicity to 5-azacytidine after 24 and 48 h of exposure (Fig. 2c and d; Table 2). Greater toxicity was recorded for exposures initiated at 1-cell stage (0.5 hpf), compared to exposures initiated at sphere stage (4 hpf) (Fig. 2c and d, *p* = 8.43e−11 and 2.11e−08 for 24 h and 48 h of exposure, respectively), with over sevenfold increase in LC50 concentrations for the later exposure period after 48 h exposure (from 14.787 to 112.997 mg/L; nominal concentrations; Table 2). A significant interaction between exposure period and toxicity was also recorded, where 0.5–48.5 hpf exposure caused steeper 0–100% mortality curve than exposures between 4 and 52 hpf (Fig. 2d, *p* = 2.04e−12). There was no significant increase in embryo mortality after 24 h of exposure when exposures were initiated at 0.5 hpf (Fig. S1c, *p* = 0.47; Table S1), but mortality continued to increase in exposures initiated at 4 hpf, with a significant difference between mortality curves for 4–28 hpf and 4–52 hpf (Fig. S1d, *p* = 0.0082; Table S1).

### Influence of pre-exposure to silver during early development on the susceptibility of larvae upon re-exposure

**Fig. 3:**
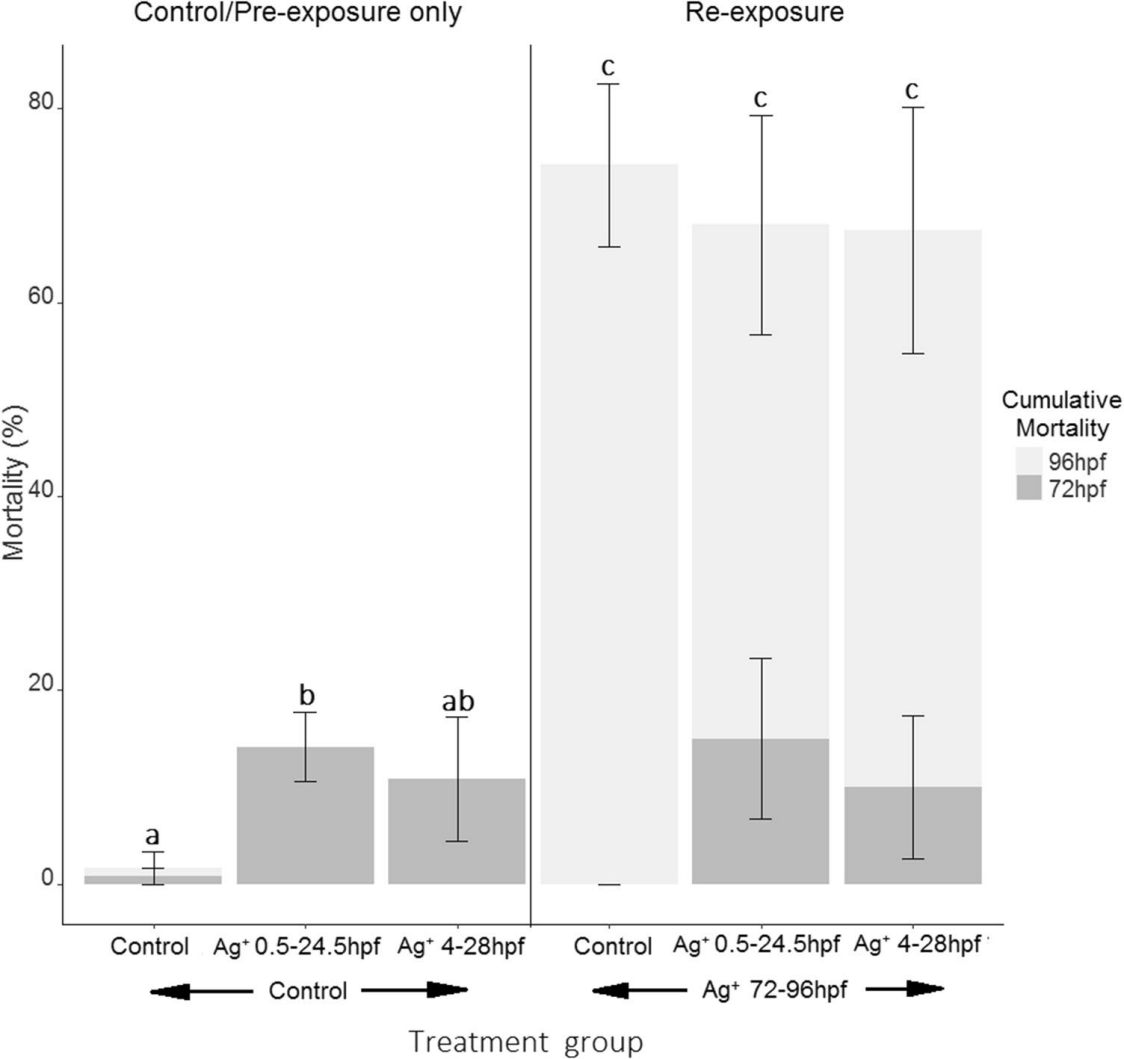
Cumulative percentage of mortality following exposure and re-exposure of embryos to silver after 72 hpf (dark grey) and 96 hpf (light grey). The first three bars describe mortality in embryos that were either kept in control conditions until 96 hpf; exposed to 30 ug/L Ag^+^ from 0.5 to 24.5 hpf; or exposed from 4 to 28 hpf. All three groups were then kept in control conditions until 96 hpf, with mortality assessed at 72 hpf (dark grey) and 96 hpf (light grey). The second three bars show mortality upon re-exposure at 72 hpf. Embryos were kept in control conditions or exposed to silver during early development and subsequently kept in clean medium until 72 hpf, as described for the previous three groups. Mortality was assessed at 72 hpf (dark grey), then all three groups were re-exposed to 30 ug/L Ag^+^ for 24 h prior to mortality assessment at 96 hpf (light grey). Treatment exposure groups indicate the conditions during each exposure period, with *n* = 6 biological replicates per treatment. Significant differences for 96 hpf cumulative mortality were determined using a Kruskal–Wallis rank-sum test, and significant pairwise differences between control, pre-exposed and re-exposed groups were determined using a Wilcoxon rank-sum test. Significant pairwise differences between treatment groups are notated by letter differences above each bar when *p* < 0.05

At 72 hpf, embryos exposed to 30 µg/L Ag^+^ for 24 h (initiated either at 0.5 or 4 hpf) showed a mortality rate below 20% (Fig. 3). Embryo mortality by 72 hpf was significantly different between control groups and embryos exposed to silver from 0.5 hpf (Fig. 3, *p* = 0.037), but not for those exposed from 4 hpf (Fig. 3, *p* = 0.596). Upon re-exposure, the percentage of mortality in exposed embryos increased significantly compared to those maintained in control conditions (Fig. 3, *p* = 0.017), but pre-exposure did not cause an alteration in cumulative mortality upon re-exposure, despite the fact that around 20% mortality had occurred in pre-exposed groups before re-exposure (Fig. 3, *p* = 0.223). Interestingly, for naïve embryos, silver toxicity was greater following 24 h of exposure when exposures were initiated after hatching (72 hpf) compared to those exposed during early development (Fig. 3, *p* = 0.014).

### Transcript profiling

**Fig. 4:**
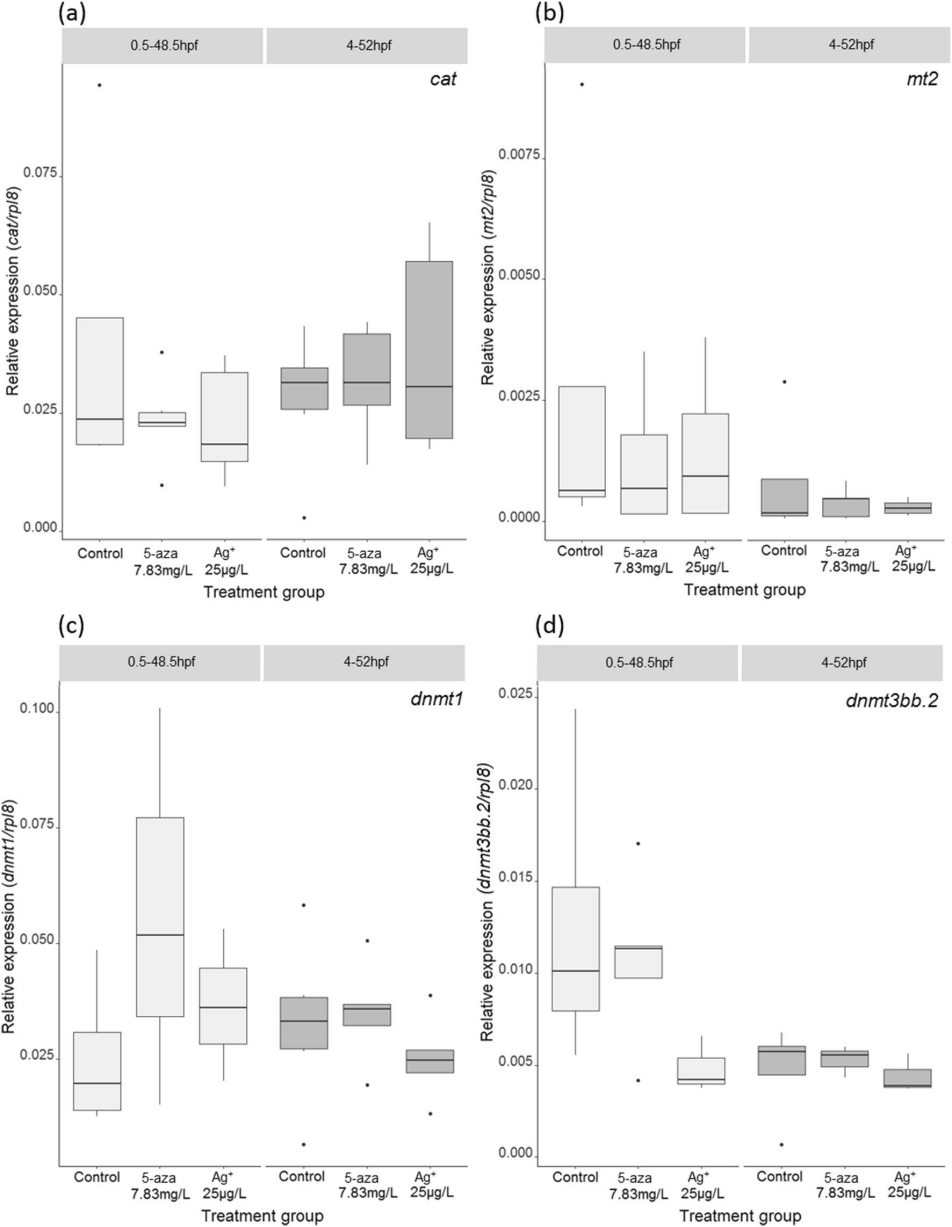
Transcript profiles for *catalase (cat), metallothionein 2* (*mt2*), *DNA (cytosine-5-)-methyltransferase 1* (*dnmt1*) and *DNA (cytosine-5-)-methyltransferase 3bb.2* (*dnmt3bb.2*) following exposure of zebrafish embryos to 5-azacytidine (abbreviated as 5-aza) and silver (added in the form of silver nitrate, Ag^+^) during the exposure period 0.5–48.5 hpf or 4-52 hpf. Embryo pools (*n* = 20 embryos per pool) were exposed to 7.83 mg/L 5-azacytidine or 25 µg/L Ag^+^. Relative expression was plotted for each embryo pool (*n* = 3–6 pools depending on the treatment group) using *rpl8* as a control gene. The Chauvenet’s criterion was applied prior to statistical analysis to remove outliers from the data. Statistics were carried out within each chemical treatment using a one way ANOVA or Kruskal–Wallis rank-sum test (depending on whether data was normally distributed). There were no significant differences between treatment groups for any of the gene transcription profiles studied

No differences in gene transcription were recorded between embryo pools kept in control conditions and those exposed to 25 µg/L Ag^+^ or 7.85 mg/L 5-azacytidine (nominal concentrations) for 0.5–48.5 hpf or 4–52 hpf for any of the gene transcription profiles studied (Fig. 4).

## Discussion

In this study, we aimed to determine whether silver toxicity was influenced by developmental stage of zebrafish, and whether pre-exposure to silver during early development caused long-lasting alterations in the susceptibility of larvae to subsequent exposures. We report that exposures initiated at the one-cell stage, and including the processes of epigenetic reprogramming and chorion hardening, caused greater toxicity than those initiated later in development. However, exposures to silver during early development did not affect the susceptibility of larvae during subsequent exposures under our experimental conditions. These findings are important to inform on the design of testing systems for chemical risk assessment using embryo models.

### Methylation inhibitor 5-azacytidine is significantly more toxic to zebrafish embryos when exposures include the epigenetic reprogramming period

We have identified a dramatic increase in toxicity (over sevenfold) for 5-azacytidine when exposures were initiated at one-cell stage (0.5 hpf) during zebrafish embryo development compared to exposures initiated during sphere stage, and after the period of epigenetic reprogramming. Embryo mortality continued to increase between 24 and 48 h for exposures initiated at 4 hpf, but no further mortalities were observed after 24 h for exposures initiated at 0.5 hpf. This may be due to its toxicity during early development when 5-azacytidine prevents re-methylation from occurring (Taylor and Jones 1982), and when embryos were most sensitive to this chemical. After 24 hpf, as its toxicity is greatly reduced, the exposure concentration is no longer sufficient to cause adverse effects at this later stage. These data corroborate the findings of Martin et al. who reported that 5-azacytidine exposure before the mid-blastula transition results in greater toxicity with over 20% of embryos showing abnormal phenotypes, but when exposures start from 3 hpf and from 6 hpf, this results in 11% and 2% abnormal phenotypes, respectively (Martin et al. 1999). Similar levels of 5-azacytidine toxicity were reported in zebrafish embryos whether the chorion had been removed before exposure or not (Martin et al. 1999), indicating that chorion permeability may not be a factor affecting 5-azacytidine toxicity. During the initial stages of zebrafish development, key cell and organ pathways are being established (Kimmel et al. 1995) and from the 16-cell stage, the epigenome of the newly formed zygote is in the process of genome-wide demethylation to remove gamete-specific methylation, followed by de novo methylation to produce an embryo-specific epigenome (Jiang et al. 2013). We hypothesise that this period of widespread de novo methylation is particularly sensitive to environmental stressors, especially those that have the potential to modify the methylome. 5-azacytidine has been shown to become incorporated into DNA and RNA, inhibiting methylation and activating specific gene regions by limiting DNA methyltransferase (*dnmt*) activity (Christman 2002). This occurs as methyltransferases irreversibly bind to 5-azacytidine residues in DNA (Taylor and Jones 1982), and this chemical is often used in the treatment of cancers where CpG islands have become methylated, inducing tumour development (Christman 2002). 5-azacytidine has been shown to mainly impact exogenous methylation including environmentally caused alterations (Bird 1992), causing global demethylation in zebrafish embryos (Bouwmeester et al. 2016; Kamstra et al. 2015a) and altered methylation sites involved in embryonic development including gastrulation and dorsal mesoderm patterning (Martin et al. 1999; Kamstra et al. 2017). Alterations to cardiomyocyte proliferation and apoptosis have also been reported in exposed zebrafish embryos, indicating widespread developmental deformities during early development exposure (Yang et al. 2019), and highlighting how 5-azacytidine demethylation is non-specific (Ceccaldi et al. 2011). Methyltransferases are of vital importance during epigenetic reprogramming (Wang and Bhandari 2019), so we propose that the dramatic increase in toxicity of 5-azacytidine observed in this study was due to the vulnerability of the methylome during early development to de novo methylation inhibition, leading to large-scale consequences to cell and organism viability. The later exposure period in this study was initiated after the somatic epigenetic reprogramming period was mostly complete (Jiang et al. 2013; Potok et al. 2013), and we predict that the epigenome is less vulnerable to disruption outside of this period. However, tissue-specific methylomes may still be affected after this period.

Investigation of some potential mechanisms of toxicity using quantitative PCR to measure target gene transcription resulted only in inconclusive results and failed to advance our understanding on how 5-azacytidine affects the embryos within our experimental setting. This could be due to multiple factors, including our choice of concentration (below that causing mortality even under the most sensitive exposure conditions), length of exposure and choice of target genes. A more comprehensive analysis using global transcription measurements, as well as an experimental design including more doses and time points, would be required to fully elucidate this question.

The exposure scenario we have designed, which included or excluded the period of epigenetic reprogramming during zebrafish embryo development, could allow for the screening and identification of chemicals that disrupt the epigenome. We hypothesise that chemicals that show greater toxicity when exposures occur during the epigenetic reprogramming period as opposed to after reprogramming and for which it is possible to demonstrate no variation in uptake dynamics are likely to disrupt the epigenome, as seen here for 5-azacyitidine. Therefore, we propose that this method could be developed to become a screening tool to identify chemicals of concern for epigenetic effects.

### Development stage at which exposures to silver are initiated determines their toxicity for zebrafish embryos

We observed a greater toxicity following silver exposures initiated at the one-cell stage compared to those initiated at blastula stage, although the differences in toxicity were far less pronounced than for 5-azacytidyne. These differences were small but very consistent across experiments (a similar difference in toxicity was also observed during the re-exposure experiments in which the first exposure caused greater mortality for embryos exposed during the earlier developmental period). Similarly, greater silver toxicity was reported for exposures initiated earlier in embryogenesis in a comparison of exposures initiated at 2, 4 and 6 hpf by 1.89-fold (2–4 hpf) and 1.13-fold (4–6 hpf), respectively (Groh et al. 2015). The continued increase in mortality across the 48 h exposure period indicates that susceptibility to silver increases in older embryos and this is corroborated by the differences in toxicity observed during the re-exposure periods (where exposures during the first day of development caused far less mortality than those conducted after hatching).

We propose three hypotheses that may explain the differences in toxicity encountered during early development. First, during early development the chorion is more permeable, as the embryo takes up water due to changes in osmotic pressure after fertilisation (Peterson and Martin-Robichaud 1982). This process has been reported to last for several hours, which could allow certain toxicants to reach embryo cells more readily, thus increasing their toxicity. Second, the embryo genome is not activated until the mid-blastula stage (Bertoldo et al. 2015), therefore, the embryo cannot successfully deploy defence mechanisms involving de novo transcription until this point. Finally, the epigenetic reprograming takes place during the first phases of embryogenesis, up to the mid-blastula stage (Jiang et al. 2013; Potok et al. 2013), and we hypothesise that organisms are more susceptible to disruption of the epigenome by environmental stressors during this highly dynamic period. All of these processes occur predominantly before 4 hpf, and either individually or in combination may explain the differences in toxicity observed.

The chorion has been shown to protect embryos during the first few days of development from a number of environmental stressors, abiotic and biotic in nature (Van Leeuwen et al. 1985; Gellert and Heinrichsdorff 2001). However, due to large changes in osmotic pressure that occur once an oocyte is spawned, the chorion swells during the first few hours of development, filling the perivitelline space through water absorption, creating a colloidal suspension of proteins (Peterson and Martin-Robichaud 1982). The increased permeability of the chorion during this time allows some chemicals to be taken up through the membrane more easily (Jezierska et al. 2009), through pores 0.6–0.7 µm wide (Kim and Tanguay 2014) or via endocytosis, as Chen et al*.* showed for graphene oxide at 2 hpf (2017). Therefore, the increase in silver toxicity observed in our study for embryos exposed earlier in development may be due to an increase in silver ions able to reach the embryo. Bӧhme et al*.* reported the presence of silver within the perivitelline space and throughout the embryo tissue when > 30 µg/L exposures began at 2 hpf (Böhme et al. 2015), and Cunningham reported no difference in silver bioaccumulation in zebrafish embryo tissue exposed from 2 hpf that had had their chorion removed to those with intact chorions (Cunningham et al. 2013). These results further attest to the notion that increased toxicity is due, at least in part, to increased silver concentrations within embryo tissues, due to chorion permeability during the first few hours of development.

Second, we hypothesised that the greater toxicity observed for exposures initiated at the one-cell stage could be due to the fact that zygotic genome activation in zebrafish does not occur until the mid-blastula stage. Before this, the embryo is largely guided by maternal gene products with synchronous cellular divisions (O’Boyle et al., 2007). It is hypothesised that transcription does not occur automatically in early developmental stages because of a lack of transcription factors and machinery, maternally loaded repressors, tightly packed chromatin and short cell cycle lengths (reviewed in Pálfy et al. 2017). Before maternal-to-zygotic transition, maternal transcripts and the regulation of translation from these transcripts, as well as regulation of protein function are the main tools for stress response in early-stage embryos (reviewed in Schulz and Harrison 2019). If the mother was exposed to the same toxicant, maternal transcripts and other factors may also include those important for the response to such a stressor that could improve survival (Plautz and Salice 2013), but this was not the case in our study. Although some regulation can occur prior to ZGA, it is far more efficient once cells can respond with rapid gene transcription to control mRNA translation (de Nadal et al. 2011). Genome activation likely plays an important role in embryo response to environmental stressors, and could be an explanation for the increase in toxicity to silver observed in our study during the earlier exposure period.

Our final hypothesis to explain the greater toxicity recorded for exposures initiated at the 1-cell stage proposes that the vulnerability of the epigenome to environmental stressors during reprogramming may explain, at least in part, the effects seen. Epigenetic alterations have been documented following environmental stressor exposure, including for silver ( Xu et al. 2018; Tai et al. 2019). Despite this, epigenetic alterations are not included in most chemical risk assessments (Mirbahai and Chipman 2014). From 16-cell to mid-blastula stage, widespread demethylation and de novo methylation occur across the genome to create a consistent zygote epigenome from the two parental germ cell genomes (Jiang et al. 2013; Potok et al. 2013; discussed above). Kamstra et al*.* highlight how important this time period is when studying environmental stressors as epigenetic modifications at this point could lead to negative adult phenotypes (Kamstra et al. 2015b), and the sensitivity of this period has been studied in mammalian systems (reviewed in Bertoldo et al. 2015). Zebrafish embryo exposure to copper during this period caused significant upregulation of 6 *dnmt3* isoforms and *mt2* (Dorts et al. 2016), and an upregulation of DNA and histone methyltransferases were observed following Atlantic cod embryo exposure to mine tailings waste (Reinardy et al. 2019). Although our study found zebrafish embryos were more sensitive to silver when the exposure window encompassed the period of epigenetic reprogramming, it is important to note that the magnitude of this difference was modest and far less than that observed for 5-azacytidine (1.2-fold compared to 7.6-fold). Therefore, we hypothesise that disruption of epigenetic pathways may contribute to the differences in silver toxicity observed, but to a far lesser extent than that observed for 5-azacytidyne.

To explore some of the potential mechanisms responsible for the differences in silver toxicity observed, we conducted transcriptional analysis of some gene biomarkers for metal toxicity (*cat* and *mt2*) and DNA methylation (*dnmt1* and *dnmt3bb.2*). No significant alterations in transcription were observed for any of the genes tested during either exposure period, similarly to that observed for the positive control 5-azacytidine, discussed above. The lack of alterations in transcription measured in our study could be due to a number of reasons, including the concentration tested (25 µg/L Ag^+^), which was below that required to cause mortality and may be insufficient to cause measurable alterations in the transcription of these biomarkers under our exposure conditions. In previous studies, a recovery from silver toxicity at the transcriptional level at 48 hpf was observed compared to earlier time points (van Aerle et al. 2013) suggesting that the lack of significant alterations observed in our study could be associated with a similar recovery process. Boyle and Goss indicated that exposure from 24–48 hpf did not cause significant metallothionein regulation, but 24 h exposures initiated at 72 or 96 hpf resulted in measurable upregulation, indicating that time since exposure initiation and developmental stage are both important factors determining transcriptional responses in a similar experimental system (Boyle and Goss 2018). Further, the lack of statistically significant effects could be due to the relatively large variability encountered, and the fact that whole body homogenates were analysed. Whole-body transcription measurements can mask individual cell transcription alterations, either through cell composition changes resulting from toxicant exposure or variation in individual cell response. Parsons et al*.* highlighted the effect of brominated flame retardants on developing zebrafish was both tissue- and developmental-stage-specific, but harder to detect in whole-body homogenates (Parsons et al*.* 2019). Finally, the target genes that were chosen for investigation may not have been altered by the exposure, but other genes and pathways may have been altered. Therefore, presence of transcriptional responses for other genes cannot be ruled out.

We demonstrate a greater toxicity for earlier exposures and propose three potential mechanisms that alone or in combination may be responsible for this observation, including chorion permeability, incomplete development of defence pathways due to an inactivated zygote genome, and epigenetic alterations, and more mechanistic studies are required to elucidate the contribution of each of these hypothesis to the alterations in toxicity reported here. Our observations demonstrate the fundamental importance of considering the time of exposure initiation when determining consequences of toxicant exposure for wild populations. In an environmentally realistic exposure scenario, gametes and embryos come into contact with toxicants immediately after they are released into the water column, so understanding sensitivity during the initial stages of development is imperative.

### Pre-exposure to silver during early development does not influence the susceptibility of zebrafish larvae upon re-exposure

We have observed no differences in mortality between naïve and pre-exposed zebrafish larvae when they were re-exposed to silver after hatching. This was the case independent of the exposure window during which embryos were pre-exposed to silver (initiated at the 1-cell stage or after epigenetic reprogramming), despite the greater toxicity observed for the earlier exposure window of 0.5–24.5 hpf. However, silver was significantly more toxic for exposures initiated at 72–96 hpf compared to those initiated at 0.5 or 4 hpf. An increase in sensitivity in hatched larvae was also reported by Böhme et al*.* for dechorinated 26–74 hpf embryos that were more sensitive to silver than 2–50 hpf chorinated embryos (Böhme et al. 2015), and has been observed for other metals, such as copper (Fitzgerald et al. 2016).

There are few reports in the literature addressing the influence of metal pre-exposure on subsequent tolerance to further exposure. Alterations in tolerance, whether due to physiological or molecular differences, can improve plasticity and have been hypothesised to lead to genetic assimilation (Badyaev 2005). Repeated exposures to copper, an essential metal, showed that two pulsed 24 h exposures at 24 and 72 hpf caused the same effects as a continuous 96 h exposure in zebrafish embryos, despite the shorter total exposure duration (Boyle et al. 2020), whereas pre-exposures to carbon tetrachloride increased tolerance to normally lethal doses in rats (Dambrauskas and Cornish 1970). Adult fish have shown increased tolerance to copper following a period of acclimation (Anadu et al. 1989), and this has been attributed to increased metallothionein expression (Bradley et al. 1985).

Our results indicate a lack of long-lasting memory in embryo cells, despite the disruption that the initial exposure inevitably had caused (demonstrated by the significant increase in mortality observed for the earlier pre-exposure group). Memory and, thus, altered response to a stressor can occur through a number of biological routes. Physiologically, cells produce proteins to respond to stressors, such as those involved in the storage or excretion of toxic chemicals (Kägi and Schäffer 1988), that can decrease response time if exposures are frequent, thereby preventing some of the toxicity. This has been shown in yeast in response to repeated salt exposure (Guan et al. 2012) and in mice, where exposure to steroids caused a significant increase in myonuclei three months after removal of the drug (Egner et al. 2013). Although protein translation in response to initial exposure is likely to have occurred, it is possible that these effects were not long-lasting or protective in our experimental system, as no increase in tolerance upon re-exposure to silver was observed. Metallothionein and other proteins active during exposure to metals have been shown to return to control concentrations in cells after a depuration period in correlation with exposure concentration (Alvarado et al. 2005). This is also supported by the observation that transcript profiles after a 48 h exposure to silver at the LC10 concentration were unaltered in our study.

Alterations in epigenetic gene regulation result in long-lasting memory within cells that can alter response to subsequent exposures independently or in conjunction with physiological effects. For example, after 14 days depuration from exposure to copper, zinc or cadmium, upregulation of metallothionein was still recorded in zebrafish gills even at exposure concentrations as low as 1 ppm (Alvarado et al. 2006). Silver could also accumulate within the embryo, particularly during the exposures initiated before chorion hardening as shown by Bӧhme et al*.* (2015), leading to embryos being exposed to a higher concentration of silver than naïve embryos upon secondary exposure, although chemical analysis would be needed to determine this. As no alteration in tolerance was observed, epigenetic alterations with consequences for susceptibility to silver are unlikely to play a major role under our experimental conditions.

Physiological and gene transcription effects are likely to have occurred to some degree during silver pre-exposure, as an increase in mortality was observed (significant for the embryos exposed from one-cell stage). Proportionally, a higher percentage of naïve embryos died during the 72–96 hpf exposure period than in pre-exposed groups that were re-exposed, although the total cumulative mortality by 96 hpf was not significantly different between groups. We hypothesise that the threshold for mortality during the second exposure was likely dictated by potential genetic differences between individuals. There is genetic variation even within laboratory strains of zebrafish (Coe et al. 2009), and we theorise that this variation causes individuals to be more or less resistant to stressors, and this variability may explain the threshold for mortality encountered after the second exposure that was common across all exposed groups.

Our observations support that exposure to silver during zebrafish embryo development, even when exposure occurs at a time of increased sensitivity, does not result in alteration of tolerance under our experimental conditions. This does not preclude the possibility that silver exposure may cause long-lasting effects or even induce differential susceptibility under different exposure conditions or for different species, and our study has the limitation of considering only one exposure duration and one concentration. However, an absence of increased tolerance to silver upon re-exposure indicates that epigenetic alterations during the reprogramming period are unlikely, and increased toxicity after embryo hatching and before chorion hardening point to chorion permeability as the primary determinant of embryo sensitivity to silver.

## Conclusion

In conclusion, we demonstrated that periods of sensitivity for silver exposure exist during zebrafish embryo development, when exposures are initiated prior to reprogramming, ZGA and chorion hardening. These three processes, alone or in combination, may be responsible for the enhanced susceptibility of zebrafish embryos to silver exposure during early development, and the evidence we present points towards chorion permeability being the most important factor. We report no alteration in susceptibility to silver following pre-exposure under our experimental conditions, independently of whether the initial exposure occurred during the most sensitive period. Our data illustrate the importance of considering how susceptibility to chemical exposure varies during early development in the design of testing systems for chemical risk assessment using embryo models.

## Electronic supplementary material

Below is the link to the electronic supplementary material.

Supplementary file1 (DOCX 146 kb)
