# Supplementary material for: Developmental exposure window influences silver toxicity but does not affect the susceptibility to subsequent exposures in zebrafish embryos
Source: Histochem Cell Biol. 2020 Oct 21;154(5):579–95. doi: 10.1007/s00418-020-01933-2 (PMC7609441; doi:10.1007/s00418-020-01933-2)
Supplement: Supplementary file 1 — Supplementary file1 (DOCX 146 kb) [file 418_2020_1933_MOESM1_ESM.docx]

**HISTOCHEMISTRY AND CELL BIOLOGY**

**Developmental exposure window influences silver toxicity but does not affect the susceptibility to subsequent exposures in zebrafish embryos**

Paige C. Robinson^1,2*^, Hannah R. Littler^1,2^, Anke Lange^1^ and Eduarda M. Santos^1,3*^

***Corresponding authors:**

Paige C. Robinson: [pr323@exeter.ac.uk](mailto:pr323@exeter.ac.uk), Eduarda M. Santos: [e.santos@exeter.ac.uk](mailto:e.santos@exeter.ac.uk), 01392 724607

**ORCID:**

**Paige C. Robinson:** 0000-0001-9038-3882

**Hannah R. Littler:** 0000-0001-7764-3790

**Anke Lange:** 0000-0003-0665-8404

**Eduarda M. Santos:** 0000-0002-4074-0121

^1^Biosciences, College of Life and Environmental Sciences, Geoffrey Pope Building, University of Exeter, Exeter, EX4 4QD, UK

^2^Centre for Environment, Fisheries and Aquaculture Science, Barrack Road, The Nothe, Weymouth, Dorset, DT4 8UB, UK

^3^Sustainable Aquaculture Futures, University of Exeter, Exeter, EX4 4QD, UK

**Acknowledgments and Funding Information**

This work is funded by a Natural Environmental Research Council iCASE Ph.D studentship (grant no. NE/P010261/1) and the Centre for Environment, Fisheries and Aquaculture Science. We thank Gregory Paull and the Aquatic Resources Centre at the University of Exeter for zebrafish husbandry support.

**Table S1** Analysis of variance models for the relationships between exposure concentrations, time at which mortalities were assessed (24 or 48h after the initiation of the exposure) and the interaction between the two variables. Zebrafish embryos were exposed to A) Silver or B) 5-azacytidine and exposures were initiated at 0.5 and 4hpf. The resulting *F* and *p* values are shown for each model. (Significance codes: ∗*p* < 0.05, ∗∗*p* < 0.01, ∗∗∗*p* < 0.001). Graphical depictions of data shown in Figure S1.

| **ANOVA** | | | | | | |
| --- | --- | --- | --- | --- | --- | --- |
| 1. **Silver** | | | | | | |
|  | **Concentration** | | **Time point for mortality assessment (24 and 48 hours)** | | **Interaction** | |
|  | *F* | *p* | *F* | *p* | *F* | *p* |
| **0.5hpf** | 198.46 | <2e-16 *** | 5.93 | 0.019 * | 1.0030 | 0.32 |
| **4hpf** | 219.47 | <2e-16 *** | 16.35 | 0.00021 *** | 1.91 | 0.17 |
| 1. **5-azacytidine** | | | | | | |
|  | **Concentration** | | **Time point for mortality assessment (24 and 48 hours)** | | **Interaction** | |
|  | *F* | *p* | *F* | *p* | *F* | *p* |
| **0.5hpf** | 381.40 | <2e-16 *** | 0.53 | 0.47 | 0.12 | 0.73 |
| **4hpf** | 67.10 | 2.93e-11 *** | 7.51 | 0.0082 ** | 1.10 | 0.30 |


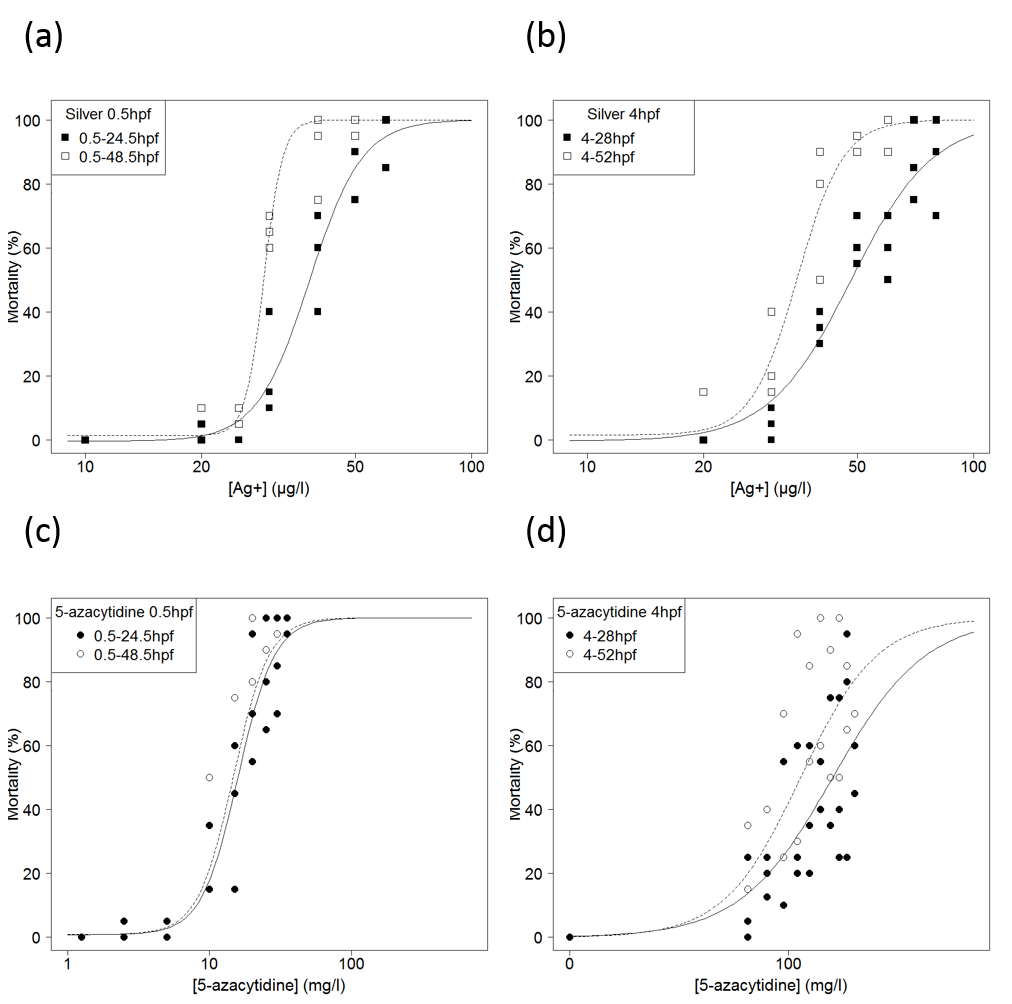


**Figure S1** Cumulative embryo mortality curves following exposure to silver or 5-azacytidine. Comparisons between mortality curves for **a)** silver exposures initiated at 0.5hpf, after 24 or 48 hours of exposure, **b)** silver exposures initiated at 4hpf, after 24 or 48 hours of exposure, **c)** 5-azcytidine exposures initiated at 0.5hpf, after 24 or 48 hours of exposure and **d)** 5-azacytidine exposures initiated at 4hpf, after 24 or 48 hours of exposure. Each point represents the percentage of mortality in one replicate dish containing 20 embryos, with three independent replicates per exposure concentration. A dose response model with four-parameter log-logistic function was fitted to produce each curve in R using the drc package. Statistical analysis of the difference between mortality curves at each exposure period are given in Table S1.
